# Unsupervised gene expression analyses identify IPF-severity correlated signatures, associated genes and biomarkers

**DOI:** 10.1186/s12890-017-0472-9

**Published:** 2017-10-20

**Authors:** Yunguan Wang, Jaswanth Yella, Jing Chen, Francis X. McCormack, Satish K. Madala, Anil G. Jegga

**Affiliations:** 1 Division of Biomedical Informatics, Cincinnati Children’s Hospital Medical Center, Cincinnati, OH USA; 2 Division of Pulmonary, Critical Care and Sleep Medicine, University of Cincinnati, Cincinnati, OH USA; 3 Division of Pulmonary Medicine, Cincinnati Children’s Hospital Medical Center, Cincinnati, OH USA; 4 Department of Pediatrics, University of Cincinnati College of Medicine, Cincinnati, OH USA; 5 Department of Computer Science, University of Cincinnati College of Engineering, Cincinnati, OH USA

**Keywords:** Idiopathic pulmonary fibrosis, Ipf, Gene expression analysis, Gene signature, IPF subtyping

## Abstract

**Background:**

Idiopathic Pulmonary Fibrosis (IPF) is a fatal fibrotic lung disease occurring predominantly in middle-aged and older adults. The traditional diagnostic classification of IPF is based on clinical, radiological, and histopathological features. However, the considerable heterogeneity in IPF presentation suggests that differences in gene expression profiles can help to characterize and distinguish disease severity.

**Methods:**

We used data-driven unsupervised clustering analysis, combined with a knowledge-based approach to identify and characterize IPF subgroups.

**Results:**

Using transcriptional profiles on lung tissue from 131 patients with IPF/UIP and 12 non-diseased controls, we identified six subgroups of IPF that generally correlated with the disease severity and lung function decline. Network-informed clustering identified the most severe subgroup of IPF that was enriched with genes regulating inflammatory processes, blood pressure and branching morphogenesis of the lung. The differentially expressed genes in six subgroups of IPF compared to healthy control include transcripts of extracellular matrix, epithelial-mesenchymal cell cross-talk, calcium ion homeostasis, and oxygen transport. Further, we compiled differentially expressed gene signatures to identify unique gene clusters that can segregate IPF from normal, and severe from mild IPF. Additional validations of these signatures were carried out in three independent cohorts of IPF/UIP. Finally, using knowledge-based approaches, we identified several novel candidate genes which may also serve as potential biomarkers of IPF.

**Conclusions:**

Discovery of unique and redundant gene signatures for subgroups in IPF can be greatly facilitated through unsupervised clustering. Findings derived from such gene signatures may provide insights into pathogenesis of IPF and facilitate the development of clinically useful biomarkers.

**Electronic supplementary material:**

The online version of this article (10.1186/s12890-017-0472-9) contains supplementary material, which is available to authorized users.

## Background

The clinical course of idiopathic pulmonary fibrosis (IPF), a chronic and fatal fibrotic lung disease, is highly variable. With a median survival of about 3 years, it ranges from a slow, steady loss of lung function over 5 or more years to a rapid progressive state and death within 1–3 years post-diagnosis. The typically slowly progressive course of IPF can be punctuated by intermittent episodes of precipitous decline in lung function termed acute exacerbation (AEIPF) [1, 2], which often lead to a new, worsened baseline of respiratory impairment. The mechanisms underlying AEIPF continue to be poorly understood [1, 3]. Further, the lack of a robust means of identifying biological heterogeneity, and selecting patient cohorts at risk for outcomes of interest continue to limit the scope and design of interventional clinical studies in IPF [1].

The current approach in IPF diagnosis is limited to clinical assessment based on imaging and histology features. Stellar attempts, however, are currently underway to develop genomic signatures and blood-specific or lung-specific biomarkers in the future [4]. Gene signatures derived from transcriptomic studies have been reported to differentiate IPF patients and from other interstitial lung diseases [5, 6] and from healthy controls [7, 8]. Comparison of gene signatures of healthy controls with ungrouped IPF patients revealed extensive genetic heterogeneity in the disease samples and differential gene expression profiles in IPF subgroups have been reported in several studies [1, 9, 10]. This demands development of computational approaches to resolve heterogeneity and identify IPF-specific transcriptomes that may help to predict disease progression. We therefore reasoned that unsupervised machine learning approaches could be applied prior to differential gene expression analysis to facilitate recognition of potential IPF subgroups with novel gene signatures that have predictive or prognostic value.

We postulated that data-driven and knowledge-based approaches using gene expression profiling of a large set of IPF/UIP cases would both allow us to identify novel patient subgroups with shared molecular characteristics and reveal novel candidate genes. Using transcriptional profiles on lung tissue from 131 patients with IPF/UIP and 12 non-diseased controls, we identified six sub-types of IPF that reflect disease severity. We have further identified molecular signatures that are capable of differentiating (a) IPF from normal controls and (b) severe from mild IPF. These signatures were subsequently validated in three independent cohorts of IPF/UIP. Finally, using knowledge-based approaches, we identified several novel candidate genes and potential biomarkers for IPF.

## Methods

### Cohort selection

We used the microarray data from the IPF cohort in the Lung Genomics Research Consortium’s (LGRC) website (http://www.lung-genomics.org; also deposited in data repository GEO - GSE47460 [11]). Among 582 subjects in dataset GSE47460, 12 had clinical and pathological designations as “controls”, and 131 had clinical and pathological diagnoses of “UIP/IPF”. We selected these 143 subjects for our cluster analysis, differential analysis, and to train classifiers. Demographic and clinical characteristics of the selected cohort are summarized in Table 1. There was no statistically significant difference in age between control and IPF patients, but there were more males in the IPF group. The predicted forced expiratory volume in one second (FEV1), forced vital capacity (FVC), and diffusing capacity of the lungs for carbon monoxide (D_LCO_) were significantly lower in UIP/IPF patients compared to those of the control group. For evaluating the classifier performance and assessing the relevance of our identified IPF sub types, we used three independent IPF cohorts (GSE24206 [8], GSE10667 [9] and GSE53845 [1]).

**Table 1:** Patient demographics and clinical characteristics of the LGRC IPF cohort

Disease Group	UIP/IPF	Control	*p* Value
Number	131	12	
Age—mean (SD)	62.6 (12.2)	64.1 (8.2)	0.5631 ^a^
%predicted FEV1 (SD)	71.37 (19.00)	94.33 (9.86)	6.3E-5 ^a^
%predicted FVC (SD)	64.78 (17.41)	91.75 (7.44)	4.3E-7 ^a^
%predicted D_LCO_ (SD)	49.33 (18.14)	97.00 (21.30)	1.1E-11 ^a^
Gender—% male	67.2	25	0.0375 ^b^

*IPF* idiopathic pulmonary fibrosis; *LGRC* Lung Genome Research Consortium; *UIP* usual interstitial pneumonia; *FVC* forced vital capacity; *FEV1* forced expiratory volume in 1 s; *DCLO* diffusing capacity of the lung for carbon monoxide; *SD* standard deviation

^a^By two-tailed Student’s t-Test

^b^By χ2 test

### Clustering, principle component analysis (PCA), and differential expression analysis

We used the Scikit-learn [12] package in Python for clustering analysis and PCA, and the limma [13] package in R for differential analysis. Data was first preprocessed by aggregating redundant transcript, log2-tranformed and median normalized across each gene, resulting an expression data matrix of 14,110 genes by 143 samples (or subjects). For PCA, the principal components were calculated using only expression data containing only IPF samples, and expression data from control and IPF patients were projected onto these principal components. Then, hierarchical clustering using the Ward linkage method on Euclidean space was performed on the transformed matrix. The number of clusters was chosen as the smallest number that allowed maximal difference in average FEV1, FVC, and D_LCO_ values among clusters. Following clustering, differential analysis was performed across IPF clusters and control with Benjamini–Hochberg false discovery rate (FDR) correction. Differentially expressed genes (DEG) were defined as those with FDR-adjusted *p*-value ≤0.05 and absolute log2 fold-change ≥1 when compared to control.

### Validation of IPF gene sets with logistic classifiers

We used the Scikit-learn package in Python to build and evaluate logistic regression classifiers to evaluate classification power of each IPF gene set. The datasets from the training and validation cohort were median normalized and scaled to (0, 1) across each gene. In order to assess classifier accuracy and reduce over-fitting, we included a 2-fold cross-validation step before training the final classifier using all samples in the training cohort. Then, classifiers were used to predict IPF status in each validation cohort. Receiver-Operating-Characteristic (ROC) curve, overall accuracy, sensitivity and specificity were used to evaluate classifier performance.

### Functional enrichment analysis and candidate gene prioritization

We used ToppFun of the ToppGene Suite [14] for functional enrichment analysis and ToppGene for candidate gene prioritization. For candidate gene prioritization, we used ‘GO: Biological Process’, ‘GO: Cellular Component’, ‘Human Phenotype’, ‘Mouse Phenotype’, ‘Pathway’ and ‘Disease’ as features to compute the similarity. The significance threshold was set as FDR-adjusted *p*-value ≤0.05. We used “known” IPF genes from the Orphanet [15] and DisGenet [16] databases as training sets to rank the differentially expressed genes in IPF and identify and prioritize novel candidate genes for IPF.

## Results

### Gene expression profiles of UIP/IPF patients are highly heterogeneous and are not consistent within clinical FVC or DLCO categories

To examine genes associated with UIP/IPF, we first performed differential gene expression analysis comparing 131 UIP/IPF patients with 12 control subjects and identified 988 differentially expressed genes. However, among these genes there were several distinctive gene expression patterns that defined distinct subsets of IPF patients (Additional file 1: Fig. S1a). To determine whether this molecular heterogeneity correlated with disease severity, we grouped UIP/IPF patients based on their available clinical FVC and D_LCO_ measurements (FVC ≥ 55% or D_LCO_ ≥ 40%: mild-to-moderate IPF; otherwise: severe IPF) and repeated the differential analysis comparing each of the phenotype-based UIP/IPF sub-groups with the control group. This resulted in 1175 and 1167 DEGs in IPF patients grouped by D_LCO_ and FVC measurements, respectively. Surprisingly, we observed that even among UIP/IPF patients within the same FVC or D_LCO_ sub group, expression of the genes was still highly variable (Additional file 1: Fig. S1b and c). This finding was further corroborated with results from principal component analysis (PCA) of all patient samples using 3657 (first quartile) most variable genes, wherein FVC or D_LCO_ subgroups could not be separated from each other by the first two principal components (Additional file 2: Fig. S2a and b). The heterogeneity within the gene expression profile of UIP/IPF patients and their poor concordance with markers of IPF severity motivated us to take an unbiased approach of clustering the IPF patients based on their gene expression profile to detect (a) potential IPF subgroups and (b) identify DEGs that correlate with lung function.

### Clustering analysis identifies UIP/IPF patient subgroups correlating with IPF-severity

**Fig. 1:**
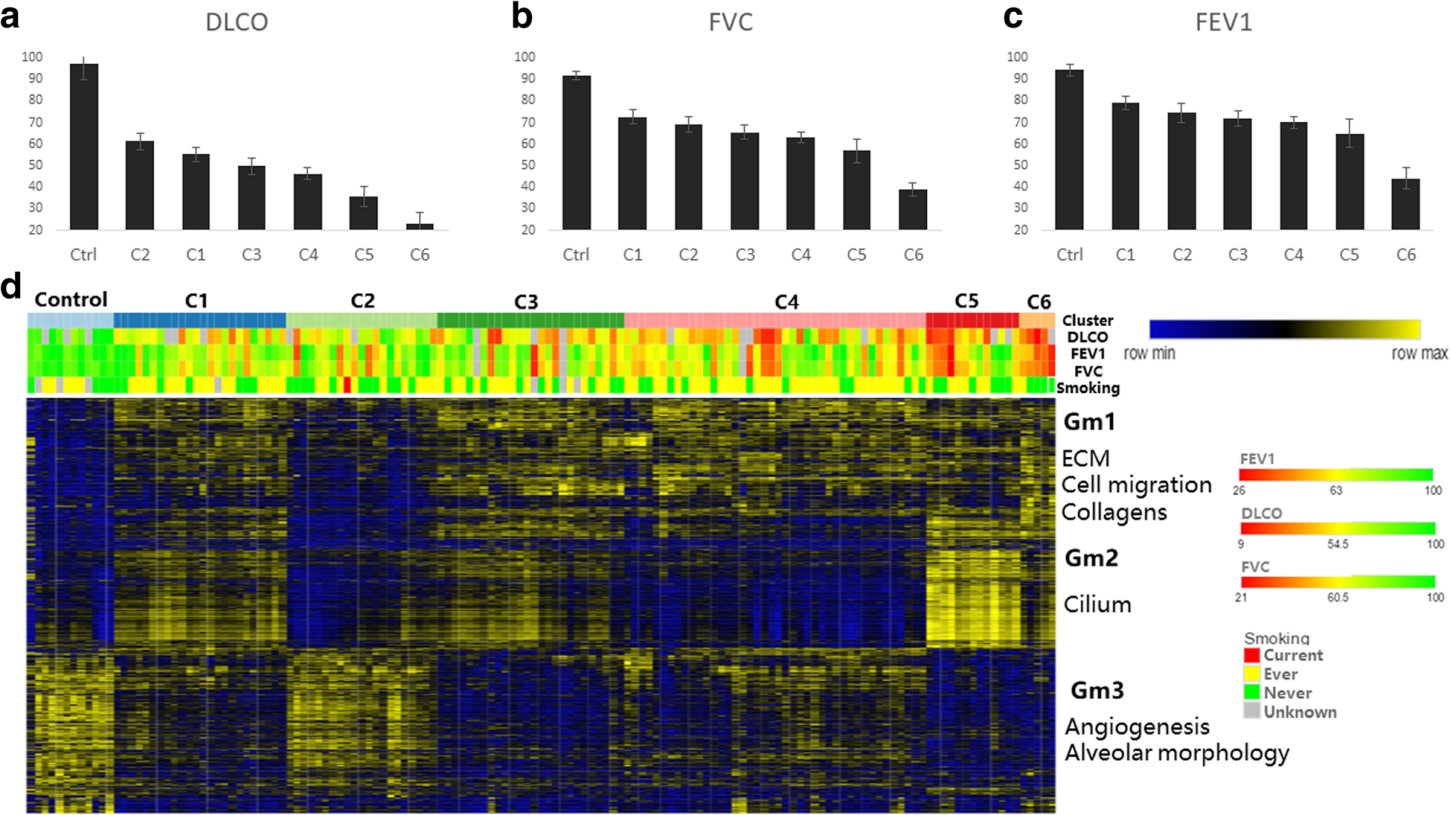
UIP/IPF patient subgroups stratified by disease severity by FEV1, FVC and DLCO have different gene expression profiles. Patient subgroups were identified using hierarchical clustering with Euclidean distance metric and Ward’s linkage (panels **a**, **b**, and **c**). Average D_LCO_ (**a**), FEV1 (**b**), and FVC (**c**) in six UIP/IPF patient subgroups. Panel d shows heat map representation of 2968 DEG (rows) in 143 control and UIP/IPF patients (columns). Genes and patients were ordered using hierarchical clustering. Color bar represents patents subgroups (1st row in heat map), D_LCO_ (2nd row), FEV1 (3rd row), FVC (4th row) and smoking status (5th row). Data are expressed as the mean ± SD. Differential expression analysis was performed using Limma. Comparison of lung function measures was carried out using two-tailed Student’s T-test

We performed ward clustering followed by PCA on the gene expression profiles of 131 UIP/IPF patients, and identified 6 distinct patient clusters (C1 through C6) (Additional file 2: Fig. S2c). Subgroups of differential IPF severity, as reflected by the average of clinical measures (D_LCO_, FEV1, and FVC), were arranged in descending order from patient clusters C6 to C1. D_LCO_ values in patient clusters C5 orC6 were significantly lower than those in C1, C2, C3, or C4, and significantly higher in patient clusters C1 or C2 than those in C3, C4, C5, or C6. On the other hand, FVC and D_LCO_ values did not differ significantly between C1 and C2, C3 and C4, or between C5 and C6 (Fig. 1a–c **;** Additional file 3: Table S1). These results suggested that the patient subgroups C1 and C2 had modest changes while C5 and C6 had a significant decline in lung function compared to control. Whereas patient subgroups of C3 and C4 exhibited intermediate changes in their lung function compared to those with mild (C1 and C2) and severe (C5 and C6) disease phenotypes based on lung function tests.

To examine transcriptomic differences between these patient clusters, we performed differential analysis comparing IPF patient clusters with control using the R package ‘limma’ and identified 2968 DEGs (Additional file 4: Table S2). Interestingly, these DEGs included nearly all DEGs identified by earlier grouping methods using (a) pooled IPF patients; (b) patients grouped based on D_LCO_ measurements; and (c) patients grouped based on FVC measurements (Additional file 5: Fig. S3) and expression of these 2968 DEGs within each of the six patient clusters was more homogenous compared to earlier methods.

Substantial differences in gene expression profiles were found between patient clusters that had similar disease severity, namely C1 vs. C2, C3 vs. C4 and C5 vs. C6 (Fig. 1d **)**. Three major gene expression modules (Gm) can be identified from the total 2968 DEGs. Gm1 was up-regulated in patient cluster C1, C3, C4, C6 and moderately in C5, Gm2 was up-regulated in patient cluster C1, C3, C5 and C6, while Gm3 was down-regulated in patient cluster C3, C5, C6 and moderately in C1 and C4. To gain functional insight into these genes, we performed enrichment analysis on them and found Gm1 was enriched in processes such as ‘extracellular matrix organization’, ‘regulation of cell migration’ and ‘collagen catabolic process’, Gm2 was enriched in processes such as ‘cilium’ and ‘cilium assembly’ and Gm3 was enriched in ‘angiogenesis’ and ‘lung alveolar morphology’. Taken together, these results show that UIP/IPF patient subgroups stratified by disease severity can be distinguished using gene expression profiles-based clustering, which in turn reveals the involvement of different molecular pathways in the pathogenesis and severity of fibrotic lung disease.

### Functional characterization of IPF subgroups

**Fig. 2:**
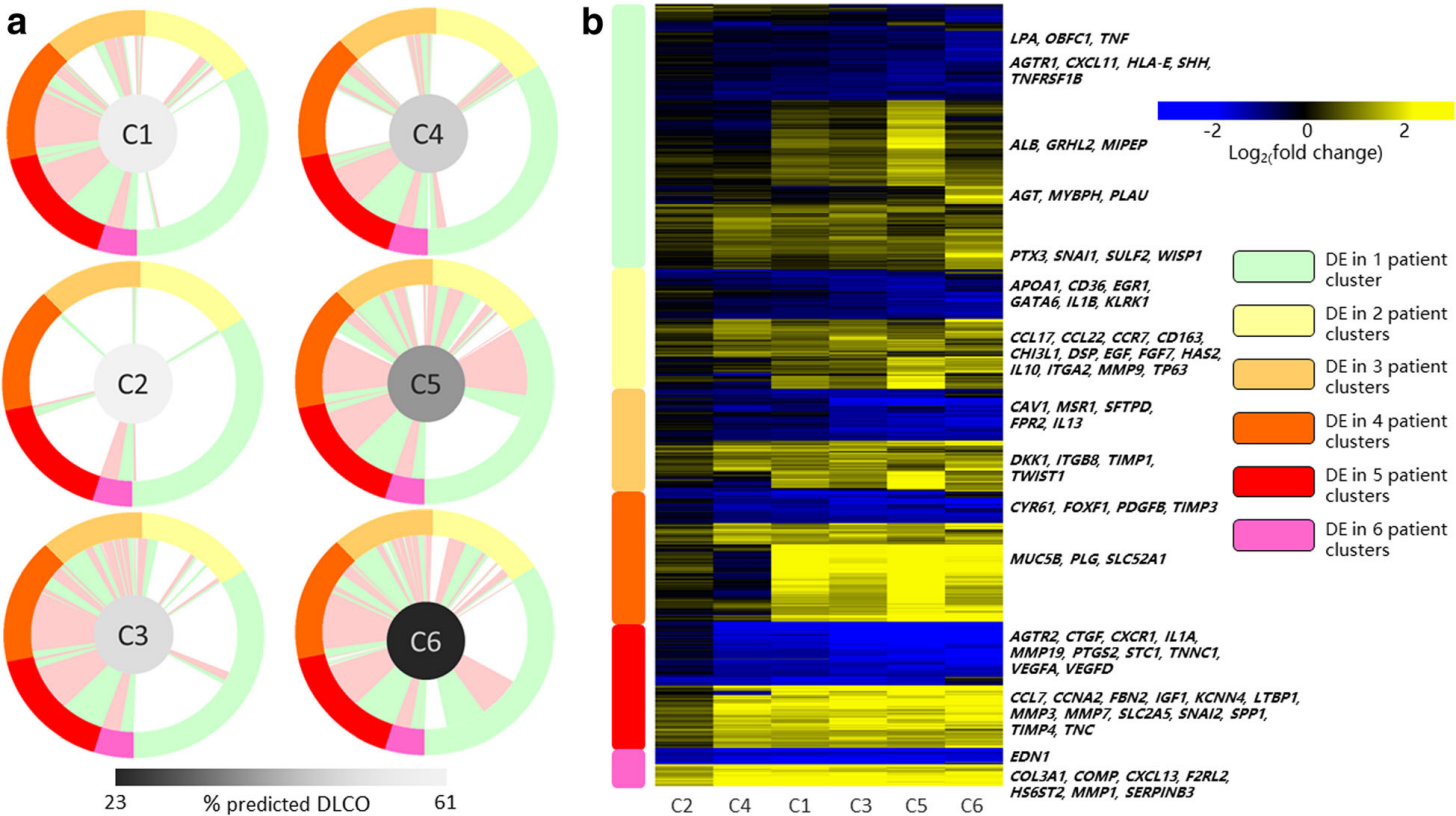
Comparison of DEGs of each patient cluster revealed genes commonly dysregulated in IPF and genes associated with severe lung function decline. DEG were divided into six groups based on the number of patient clusters where a gene was differentially expressed. Panel **a** shows schematic representation of 2968 DEG in six IPF patient clusters. DEGs along with their group designation are shown in the same order along the outer rim of each circular plot. The center of each circular represents patient cluster with the color intensity representing average % predicted D_LCO_ in that cluster. Each colored edge (red: up-regulated; green: down-regulated) from a patient cluster to a gene in the rim indicates differential expression of that gene in the connected patient cluster. Panel **b** is a heat map representation of the 2968 DEG. Up- or down- regulated genes in each group that are involved or implicated in IPF were highlighted

**Fig. 3:**
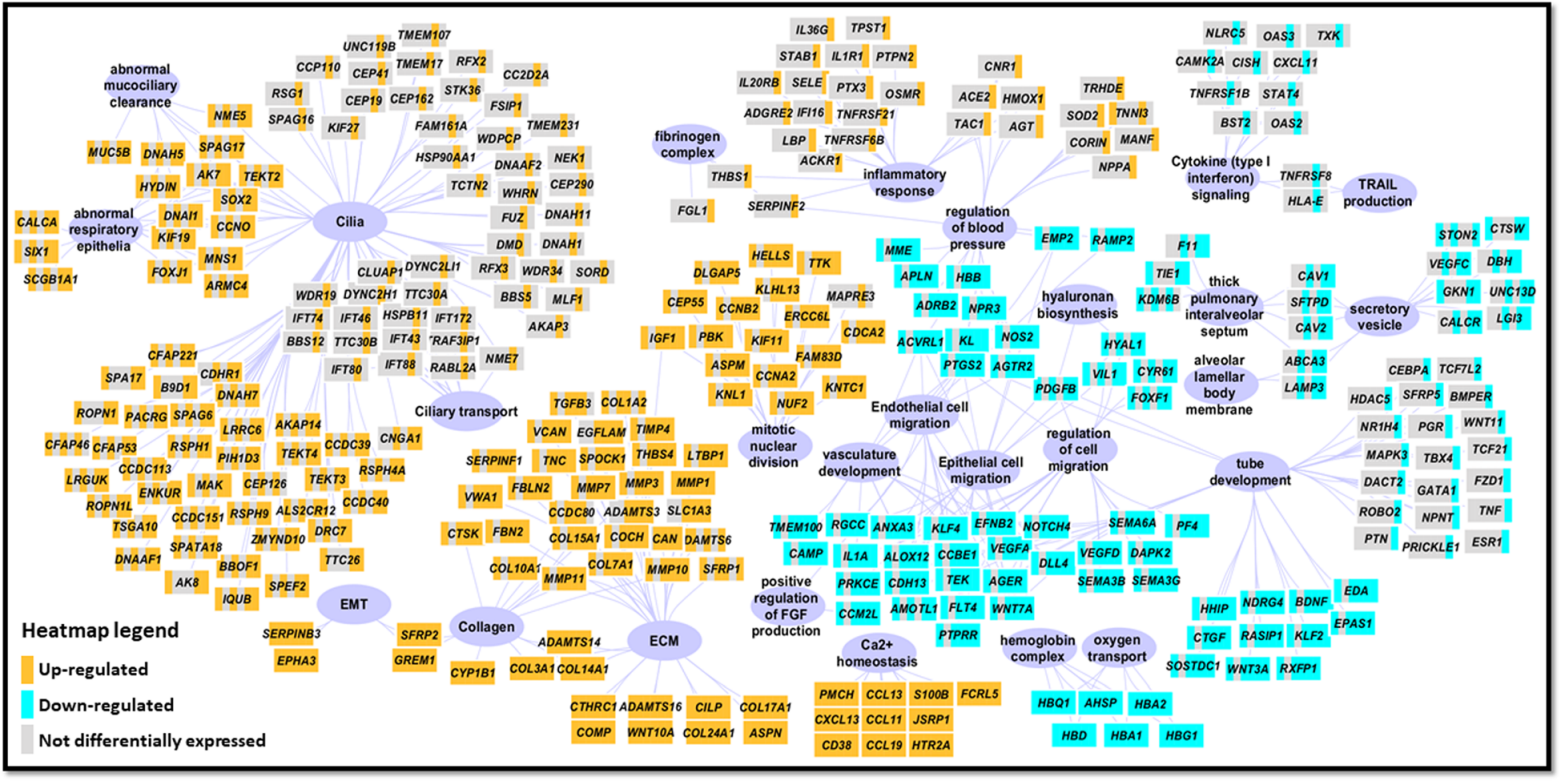
Enriched biological processes in each gene category revealed commonly and high-severity-associated biological pathways perturbed in IPF. Selected enrichment terms derived from gene lists in DEG groups were shown. Connection from a gene (rectangular node) to a biological process (purple oval node) indicates involvement of that gene in the connected process. Differential expression status of a gene in each patient subgroup was shown as a mini heat map (orange: up-regulation; turquoise: down-regulation; gray: not differentially expressed, patient subgroup order: C1, C2, C3, C4, C5 and C6). Network was made in Cytoscape 3.5, and layout was performed using AllegroLayout v2 Professional with manual curation

The number of DEG found in each of the patients’ clusters ranged from 262 genes (patient cluster C2) to 2117 genes (C5). About 34% of the total DEGs were unique to one patient subgroup while the remaining were found to be overlapping with the others (Fig. 2a and b **)**. All IPF subgroups shared a set of 145 DEGs, which were named the *IPF core* gene set. Among them, genes involved in ‘proteinaceous extracellular matrix’ and ‘regulation of epithelial to mesenchymal transition (EMT)’ were up-regulated, while hemoglobin genes such as *HBA1, HBA2, HBD, HBG1* and *HBQ1* were down-regulated (Fig. 3 **)**. The most severe patient subgroups C5 andC6 shared DEGs in C1 and C3, which were enriched in processes including ‘mitotic nuclear division’, ‘cilium assembly’, ‘epithelial/endothelial migration’ and ‘tube development’. Patient clusters C5 andC6 also uniquely expressed 840 DEG, with 448 genes in C5 and 392 genes inC6. Subsets of C5 unique genes were enriched in pathways such as ‘cilium assembly’ and ‘tube development’, which were also perturbed in less-severe IPF subgroups C1 and C3. This suggests a potential IPF progression path of C1➔C3➔C5 characterized by increasing expression of cilium-associated genes, and is in consistent with a previous study that reported a positive correlation of cilium-associated gene expression and increased IPF severity [10]. TheC6-specific gene set included inflammatory response genes such as *HMOX1*, *IL1R1*, *IL20RB*, *IL36G*, *SELE*, *SERPINF2*, *TNFRSF21* and *TNFRSF6B,* but not genes enriched in cilium-associated pathways (Fig. 3 **)**. This suggests IPF can alternatively progress via up-regulating inflammation genes without further up-regulation of cilium-associated genes, and is consistent with a recent report showing increased inflammation in rapid progressive IPF [17].

### Validation of IPF subgroups with independent IPF cohorts

To further validate and assess the relevance of our identified IPF subgroups, we used three independent IPF cohorts (GSE24206, GSE10667 and GSE53845). By utilizing multiple testing datasets, we determined if the gene sets reveal key differences in gene expression between IPF and normal, and between severe IPF (explant) and usual IPF (biopsy).

**Fig. 4:**
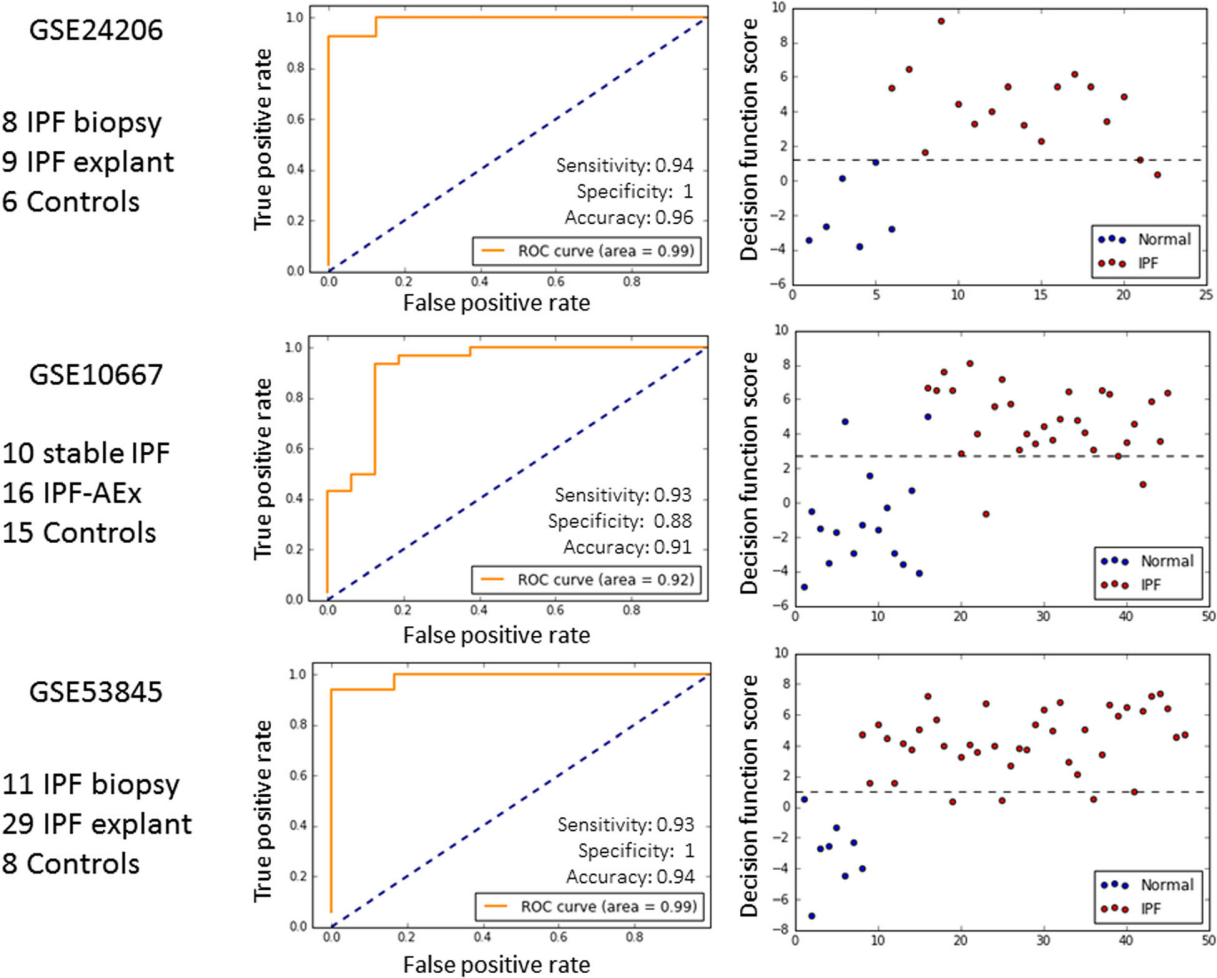
The core IPF gene set robustly differentiated IPF patients from normal controls in three independent validation cohorts. Logistic regression models were trained on the core IPF gene sets using the training cohort with 2-fold cross-validation, and tested with each validation cohort. The decision threshold was set to provide at least 90% sensitivity for IPF discovery. ROC curves were shown in the left column, and classification scatter plots of IPF and control samples were shown in the right column

Out of 145 genes in the core IPF gene set (Additional file 6: Fig. S4), 133 were found in all three validation cohorts. We used the LGRC dataset with 12 controls and 131 IPF patients as the training set, and trained a logistic regression classifier for classification of IPF patients. Then, IPF status (normal or IPF) was predicted by using the classifier on each validation dataset, where the decision threshold was set to provide at least 90% sensitivity. The ROC curve, sensitivity, specificity, and overall accuracy in each of the validation datasets are shown in Fig. 4 **.** Specificity, sensitivity and accuracy in all three validation datasets were >90%. Similarly, we validated the C5 andC6 unique gene sets (Additional file 7: Fig. S5). The C5 unique gene set (448 genes) performed poorly in differentiating severe IPF from usual IPF in all validation datasets (data not shown). Hence, this gene set was not considered for further analysis. However, although classifiers built on theC6 unique gene set failed to distinguish AEIPF from IPF, they could moderately differentiate IPF explant from IPF biopsy, indicating the unique gene expression profile of patient clusterC6 were also present in severe, end-stage IPF. Taken together, these results demonstrate that the core IPF gene set is a robust gene signature to separate IPF from control. The advanced IPF gene set (c6 unique gene set) on the other hand can differentiate advanced IPF, but not AEIPF, from stable IPF.

### Functional prioritization of novel IPF-associated genes

To identify genes that were most functionally relevant to biologic processes perturbed in IPF, we employed a systems biology approach to rank each gene in the core and advanced gene sets based on their functional similarity to one of the two training sets comprising genes known or implicated to be involved in IPF [15, 16] (Additional file 8: Table S3) and their gene expression fold change in IPF compared to control. Specifically, functional similarity was calculated using the ToppGene Suite’s gene prioritization tool [14]. Genes in the core and advanced IPF gene sets that were also in the training set (“known” IPF genes) were removed from ranking. The remaining 133 and 382 genes from the core and advanced gene sets respectively were then ranked separately based on either similarity score or fold change compared with normal. The rankings of each of the genes were aggregated using the rank product method [18]. Top 10% ranked genes in the advanced and core IPF gene set are shown in Table 2. Twenty-two of these genes had been shown to be differentially expressed in IPF patients compared with healthy control or involved in IPF pathogenesis. Enrichment analysis of the novel candidates showed these genes were involved in pathways often perturbed in IPF. For example, up-regulated genes in the advanced set such as *SERPINF2, MMP14, DMP1 and CTSL*, were enriched in ‘extracellular matrix organization’. On the other hand, genes involved in leukocyte activation such as *BLM, RAG1, PRKCZ, LBP, and MMP14* were only present in the advanced gene set.

**Table 2:**
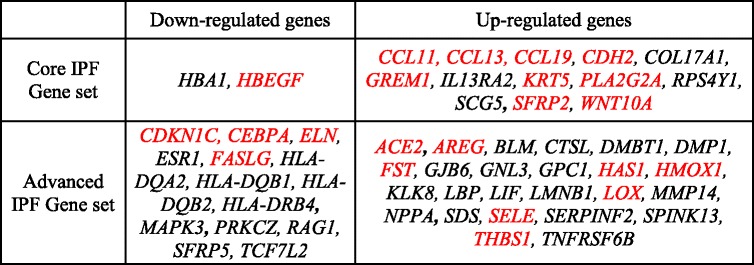
Top 10% prioritized genes in the core and advanced IPF gene sets

Genes in red font color represent genes that have been reported to be related to IPF (PubMed-based literature mining). Genes in core IPF and advanced IPF (patient clusterC6) gene sets were prioritized using ToppGene application of the ToppGene Suite and their absolute fold change in IPF compared to controls

### Prioritization of putative bronchoalveolar lavage fluid biomarkers for IPF

Among the genes in the core IPF gene set, 60 of them encode secreted proteins (based on Uniprot [19] annotation) or were previously found in bronchoalveolar lavage fluid (BALF). Given these genes’ classification power and their potential clinical utility, we decided to prioritize candidate IPF BALF biomarkers among them. We first ranked these genes based on magnitude of the coefficients from a logistic regression model. Then, we built a series of logistic regression models, each trained on up to 50 top ranked genes, to determine the threshold for marker selection (Additional file 9: Fig. S6). In the end, we identified 11 putative biomarkers, including *HMGCS2, CHL1, DAO, CRTAC1, EDN1, WNT10A, HBEGF, IL6, CCK, EPHA3* and *SEMA3E,* in the core IPF gene set (details in Additional file 10: Table S4) which is the smallest biomarker set that allowed >0.8 specificity and 0.9 sensitivity in distinguishing IPF from healthy control. Notably, three core IPF markers, *HMGCS2*, *CHL1* and *SEMA3E*, were also differentially expressed compared with control in BALF of bleomycin-treated mouse and their direction of dysregulation was consistent with our study [20].

## Discussion

In this study of patients with UIP/IPF, we stratified subgroups based on lung function measures and applied unsupervised analysis on gene expression data. Genes enriched in cilium or lung alveolar morphology were expressed at different levels in two distinct transcriptomic profiles from patient clusters with moderate disease (cluster average D_LCO_: 40–60%), but not severe disease (cluster average D_LCO_: <40%). Comparison of DEG from each patient cluster revealed additional gene signatures that robustly differentiated IPF from normal lung, and advanced IPF from usual IPF. Finally, using knowledge-based approaches, we identified several novel gene candidates and potential BALF biomarkers for IPF.

The uniqueness of current study is that we used unsupervised, data-driven approaches to discover potential subgroups within IPF patient samples prior to extracting IPF-specific gene signatures, which allowed us to identify genes commonly involved in IPF or only associated with advanced IPF. In contrast, gene signatures of previous studies were all derived from comparing pooled IPF samples with healthy controls [5–9]. As a result, we identified additional 1981 DEG along with genes discoverable without incorporating clustering steps prior to differential analysis, and 382 out of 392 advanced IPF genes were among the additional genes.

Our results indicate that gene expression profiles from IPF patients are heterogeneous. Grouping patients according to lung function measures such as FVC and D_LCO_ reduced such heterogeneity and allowed discovery of more DEG. However, different gene expression profiles could still be found within the lung function group defined based on FVC or D_LCO_ measurements, and several genes were expressed at similar levels in different patient groups. Gene expression heterogeneity not yet explainable by lung function measures suggested activation of disease-driving pathways that could potentially be informative in efforts to improve the therapeutic response and outcome. On the other hand, genes expressed at comparable levels across patient subgroups of different severity suggest potential involvement of these genes and linked biological processes in distinct stages of IPF. In this study, we validated these clusters by cross referencing with clinical data to avoid generating clusters that are less relevant clinically. Clustering patients based on gene expression prior to differential analysis may also help to circumvent some of the limitations we encountered.

A recent study reported that cilium-associated genes were associated with more extensive microscopic honeycombing in IPF patients, although no difference in lung function measures were found in patient groups defined by these genes [10]. Our results are consistent with these data in that we found that cilium-associated genes are most highly expressed in patient cluster C5 with more severe IPF. These genes include *MUC5B* and *DSP* which were known to be involved in IPF [10, 21]*,* matrix metalloproteinases that are implicated in IPF such as *MMP1, MMP3* and *MMP7* [22], and collagens involved in ECM organization. However, cilium-associated genes were also highly expressed, although to a less extent, in less-severe patient clusters, C1 and C3. More importantly, patient cluster C4 with low cilium-associated gene expression had D_LCO_ values that were comparable to those of C3, suggesting potential additional driver genes underlying IPF severity.

Our analyses revealed novel IPF associated genes and biomarkers. Among the 55 prioritized genes, 22 were previously shown to be dysregulated in IPF or involved in IPF pathogenesis. For example, up-regulated expression of candidate genes including *CTHRC1, CTSE, GREM1, NELL1* and *PLA2G2A* in the core set, and *AREG, FST, LOX, THBS1* and *SELE* in the advanced set, were also found to be increased in IPF animal models or in human IPF patients [7, 23–27]. On the other hand, *ACE2*, *SFRP2* and *WNT10A* were known to be associated with fibrosis in IPF animal models and survival in IPF patients [28–30]. The presence of these genes in the candidate list supports the validity and robustness of our prioritization although further studies are needed to validate the remaining novel candidate genes identified. In addition to novel candidate IPF genes, we also identified putative BALF biomarkers that can potentially differentiate IPF patients from healthy normal volunteers. The high consistency of the expression of these biomarker genes with their corresponding protein expression in BALF [20] suggest that classifiers built on them could achieve comparable predictive power observed in our study. Thus, our biomarker list may inform future efforts to identify diagnostic, predictive and prognostic biomarkers in BALF that could obviate the need for more invasive diagnostic maneuvers and be used in decision making for IPF care.

## Conclusions

In conclusion, our results show that discovery of robust gene signatures for IPF diagnosis can be greatly facilitated through integration of unsupervised and systems biology approaches. Findings derived from gene signatures may provide insights into pathogenesis of IPF and facilitate the development of clinically useful biomarkers.

## Additional files

Additional file 1: **Figure S1.** Gene expression profiles in lung tissues taken from IPF patients were highly heterogeneous. IPF samples were pooled (a) or grouped based on FVC (b) or D_LCO_ (c). Differentially expressed genes were then extracted from each condition with FDR-adjusted *P*-value cut-off at 0.05 and fold-change cut-off at 2. Genes (rows) and samples (columns) were ordered using hierarchical clustering with Pearson correlation distance metric and complete linkage. (TIFF 1174 kb)

Additional file 2: **Figure S2.** Principal Component analysis (PCA) plot characterized separation of IPF sample by three grouping methods. Distribution of IPF samples along the first two principal components derived from top 25% most variant genes are shown, and sample grouping were based on FVC (a), D_LCO_ (b), or Ward clustering(C). Each point represents an IPF sample. (TIFF 282 kb)

Additional file 3: **Table S1.** Comparison of FVC, FEV1, D_LCO_ and age in six IPF patient subgroups. (XLSX 11 kb)

Additional file 4: **Table S2.** List of all DEG compared with health control. (XLSX 339 kb)

Additional file 5: **Figure S3.** Unsupervised clustering followed by differential analysis recovered almost all the DEG identified by other methods and discovered additional DEG. Comparison of differentially expressed gene identified based on different IPF patient grouping methods. Pooled-IPF, IPF patients were not divided into subgroups; FVC or DLCO grouping, IPF patients were divided into subgroups based on the FVC or DLCO categories, respectively; Clustering, IPF patients were divided into subgroups using PCA and Ward clustering. (TIFF 151 kb)

Additional file 6: **Figure S4.** Heat maps of Core and advanced IPF gene set. 145 core IPF genes (a) and 392 advanced IPF genes were ordered using hierarchical clustering with Pearson correlation distance and complete linkage method. Patients (columns) were ordered in the same way in each heat map. (TIFF 770 kb)

Additional file 7: **Figure S5.** The advanced IPF gene set could differentiate end-stage IPF but not AEIPF from usual IPF. Logistic regression models were trained on the advanced IPF gene sets using the training cohort, and tested using each validation cohort. The decision threshold was set to provide at least 90% sensitivity for IPF discovery. ROC curves were shown in the left column, and classification scatter plots of IPF and control samples were shown in the right column. (TIFF 163 kb)

Additional file 8: **Table S3.** List of genes used as training set for ToppGene prioritization. (XLSX 20 kb)

Additional file 9: **Figure S6.** Performance of Logistic Regression Classifier build on up to 50 top ranked putative BALF biomarkers. Putative BALF biomarkers were ranked based on the magnitude of their decision function coefficient derived from a logistic classifier trained using the training cohort. A series of logistic classifiers trained on up to 50 top ranked genes using the training cohort were tested using each validation cohort. The decision threshold was set to provide the highest prediction accuracy. (TIFF 78 kb)

Additional file 10: **Table S4.** List of potential BALF biomarkers in the core IPF gene set. (XLSX 12 kb)

## Abbreviations

DEG: Differentially expressed gene
D_LCO_: Diffusing capacity of the lungs for carbon monoxide
FDR: Benjamini–Hochberg false discovery rate
FEV1: Forced expiratory volume in the first one second
FVC: Forced vital capacity
IPF: Idiopathic pulmonary fibrosis
PCA: Principal component analysis
SD: Standard deviation
