# Supplementary figures and images for: Unsupervised gene expression analyses identify IPF-severity correlated signatures, associated genes and biomarkers
Source: BMC Pulm Med. 2017 Oct 20;17:133. doi: 10.1186/s12890-017-0472-9 (PMC5649521; doi:10.1186/s12890-017-0472-9)

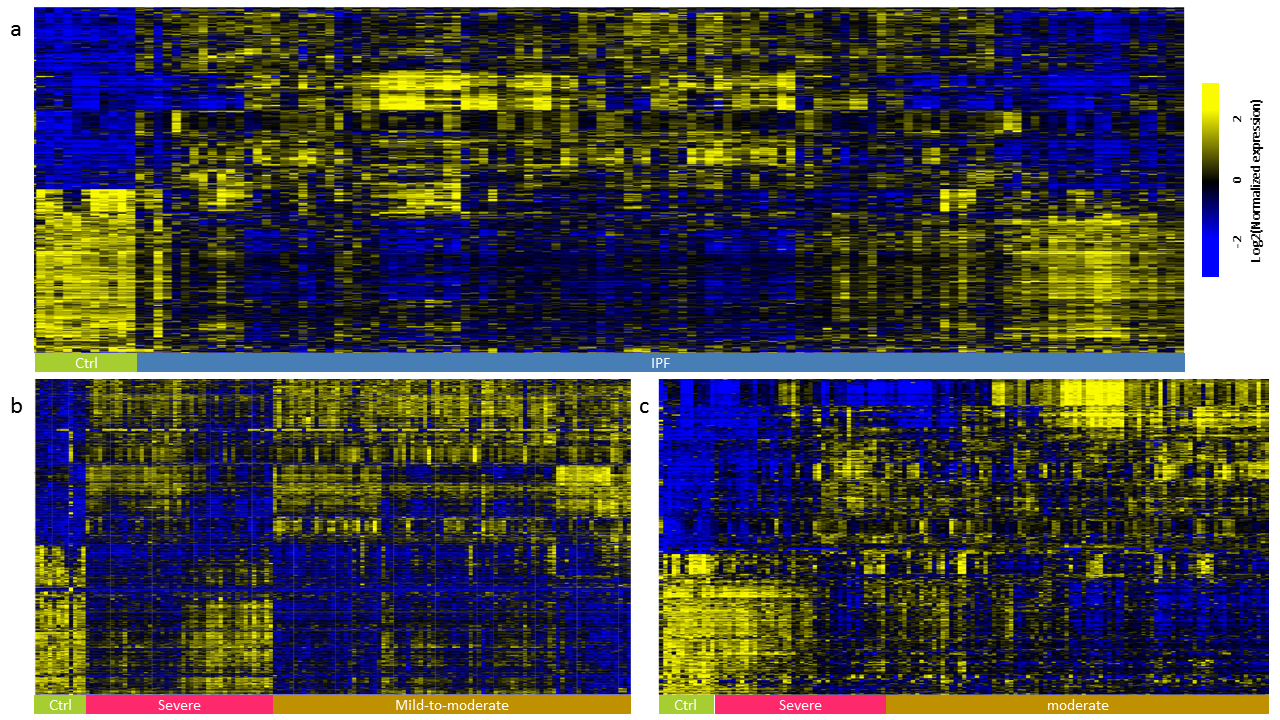

Supplement: Supplementary file 1 — Figure S1. Gene expression profiles in lung tissues taken from IPF patients were highly heterogeneous. IPF samples were pooled (a) or grouped based on FVC (b) or DLCO (c). Differentially expressed genes were then extracted from each condition with FDR-adjusted P-value cut-off at 0.05 and fold-change cut-off at 2. Genes (rows) and samples (columns) were ordered using hierarchical clustering with Pearson correlation distance metric and complete linkage. (TIFF 1174 kb) [file 12890_2017_472_MOESM1_ESM.tif]

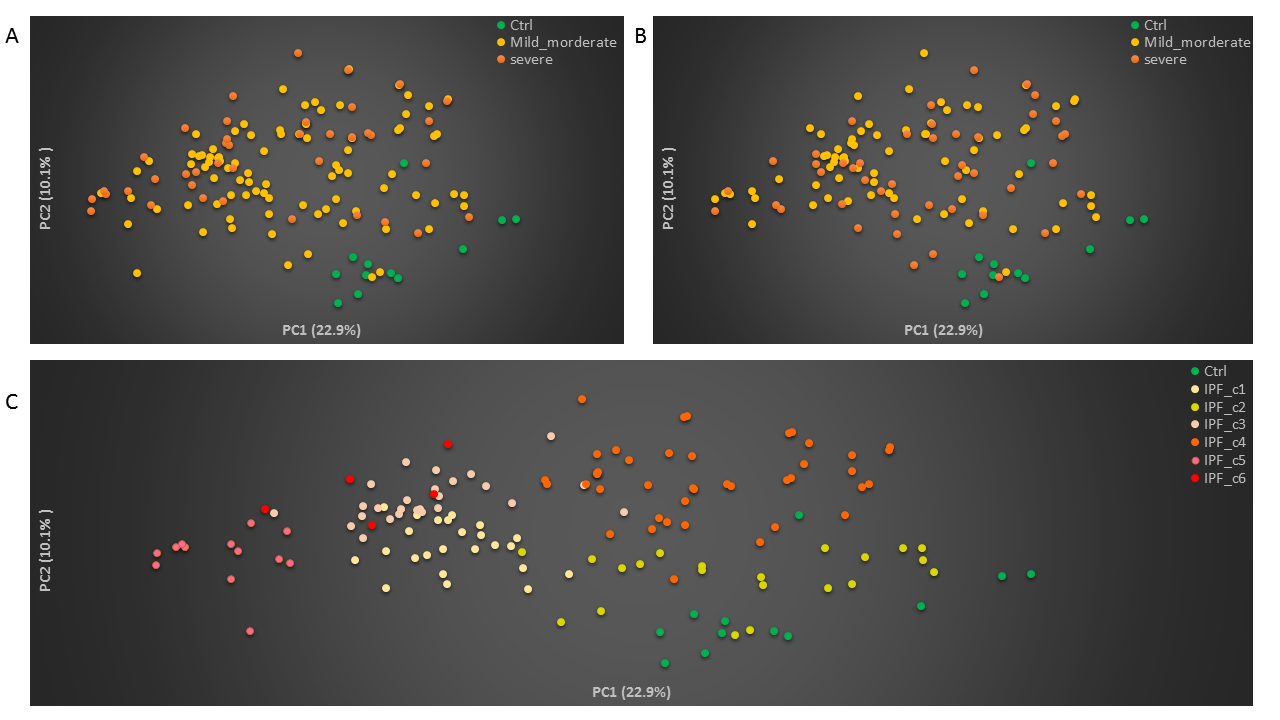

Supplement: Supplementary file 2 — Figure S2. Principal Component analysis (PCA) plot characterized separation of IPF sample by three grouping methods. Distribution of IPF samples along the first two principal components derived from top 25% most variant genes are shown, and sample grouping were based on FVC (a), DLCO (b), or Ward clustering(C). Each point represents an IPF sample. (TIFF 282 kb) [file 12890_2017_472_MOESM2_ESM.tif]

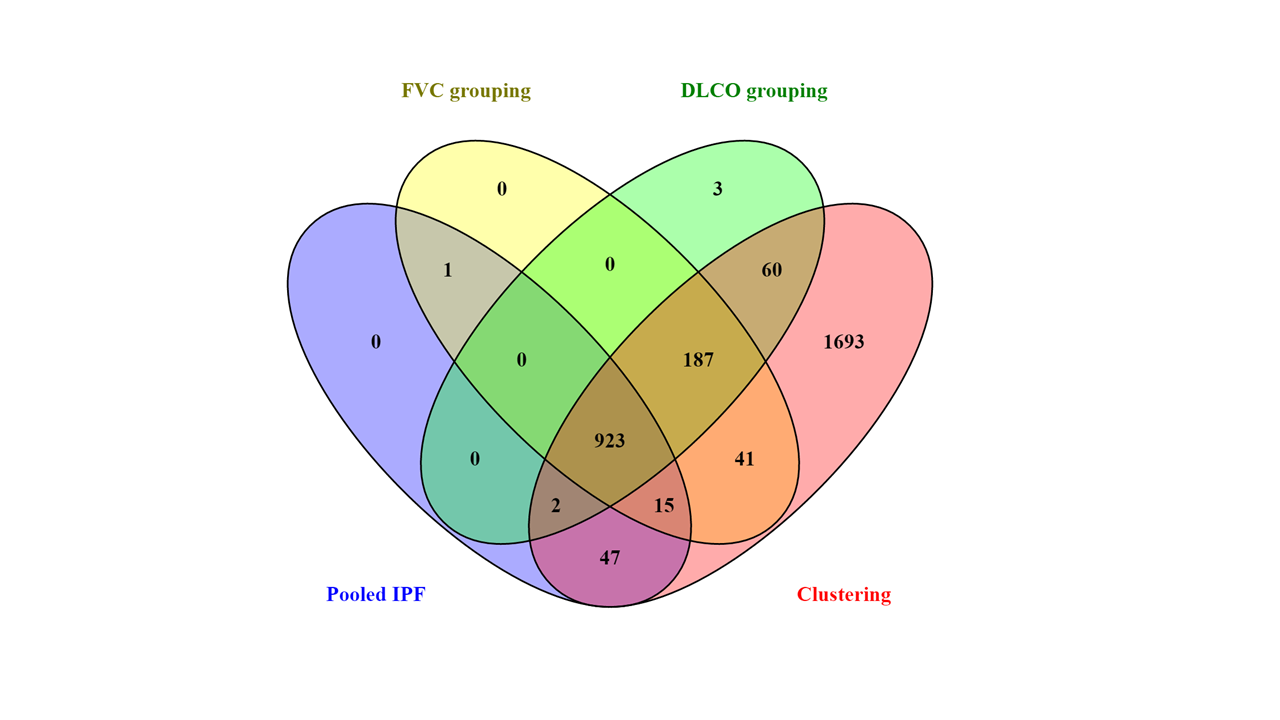

Supplement: Supplementary file 5 — Figure S3. Unsupervised clustering followed by differential analysis recovered almost all the DEG identified by other methods and discovered additional DEG. Comparison of differentially expressed gene identified based on different IPF patient grouping methods. Pooled-IPF, IPF patients were not divided into subgroups; FVC or DLCO grouping, IPF patients were divided into subgroups based on the FVC or DLCO categories, respectively; Clustering, IPF patients were divided into subgroups using PCA and Ward clustering. (TIFF 151 kb) [file 12890_2017_472_MOESM5_ESM.tif]

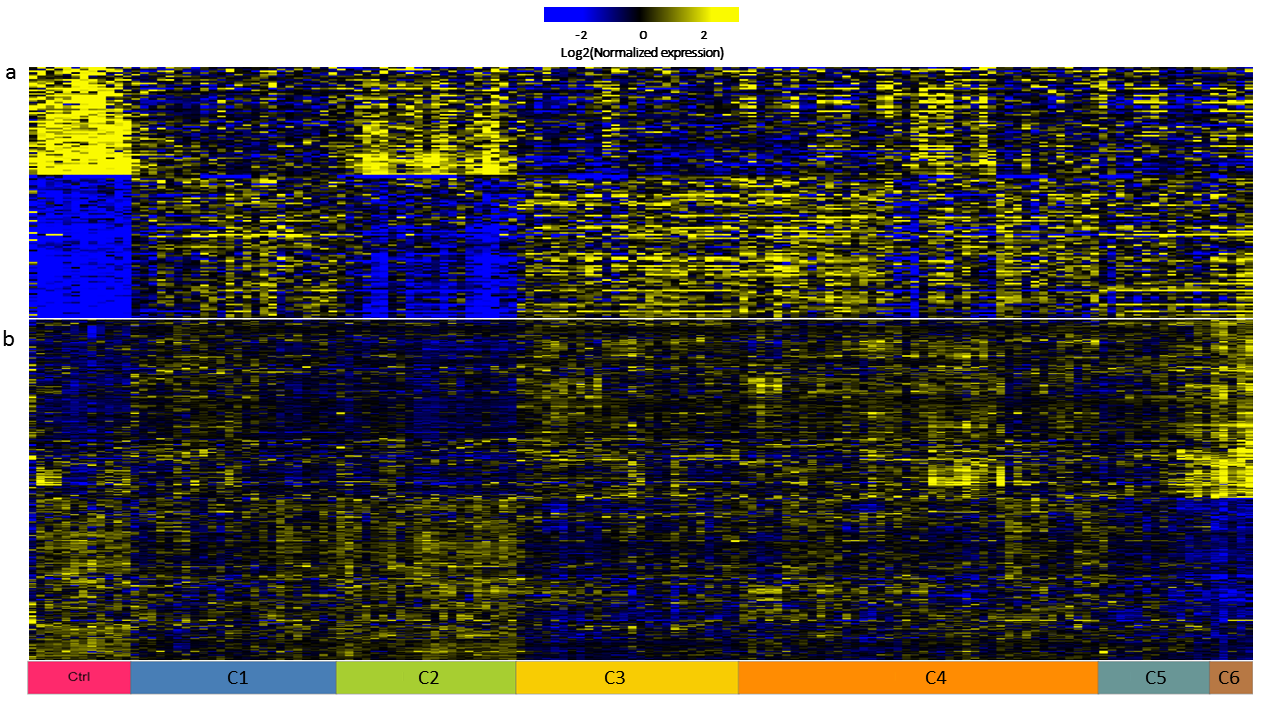

Supplement: Supplementary file 6 — Figure S4. Heat maps of Core and advanced IPF gene set. 145 core IPF genes (a) and 392 advanced IPF genes were ordered using hierarchical clustering with Pearson correlation distance and complete linkage method. Patients (columns) were ordered in the same way in each heat map. (TIFF 770 kb) [file 12890_2017_472_MOESM6_ESM.tif]

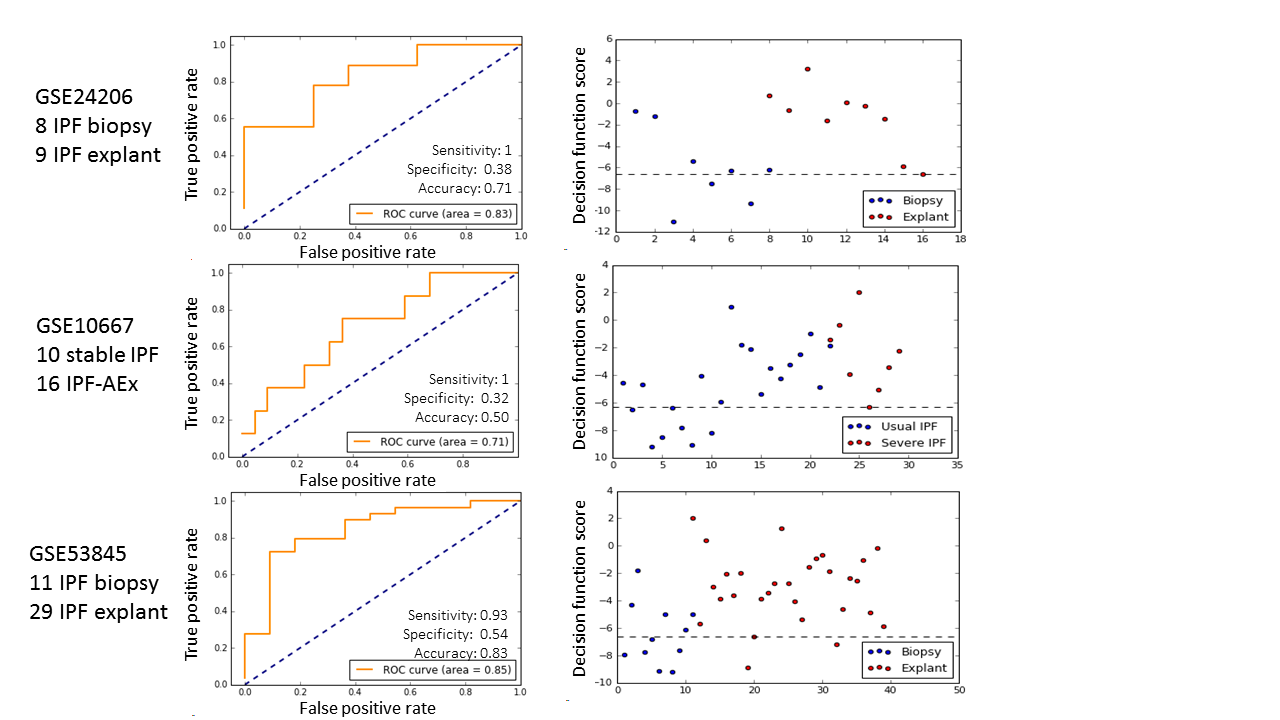

Supplement: Supplementary file 7 — Figure S5. The advanced IPF gene set could differentiate end-stage IPF but not AEIPF from usual IPF. Logistic regression models were trained on the advanced IPF gene sets using the training cohort, and tested using each validation cohort. The decision threshold was set to provide at least 90% sensitivity for IPF discovery. ROC curves were shown in the left column, and classification scatter plots of IPF and control samples were shown in the right column. (TIFF 163 kb) [file 12890_2017_472_MOESM7_ESM.tif]

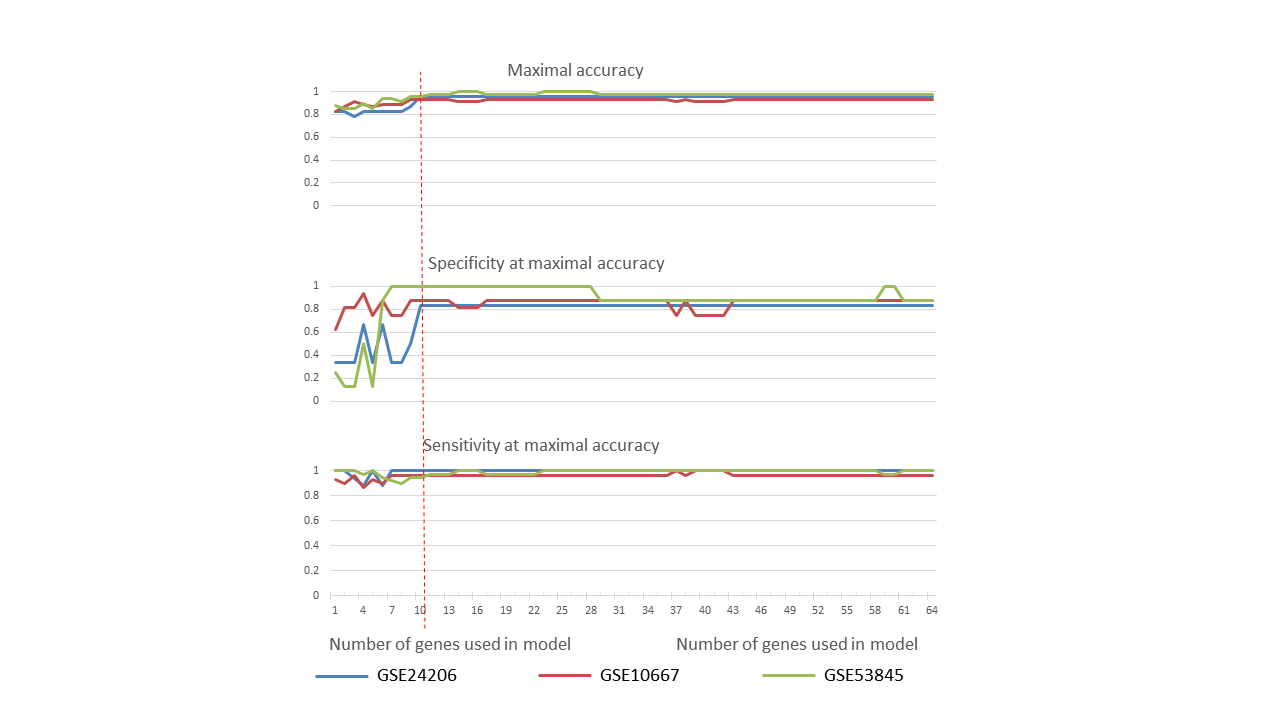

Supplement: Supplementary file 9 — Figure S6. Performance of Logistic Regression Classifier build on up to 50 top ranked putative BALF biomarkers. Putative BALF biomarkers were ranked based on the magnitude of their decision function coefficient derived from a logistic classifier trained using the training cohort. A series of logistic classifiers trained on up to 50 top ranked genes using the training cohort were tested using each validation cohort. The decision threshold was set to provide the highest prediction accuracy. (TIFF 78 kb) [file 12890_2017_472_MOESM9_ESM.tif]
